# Intraspecific Trait Variation and Phenotypic Plasticity Mediate Alpine Plant Species Response to Climate Change

**DOI:** 10.3389/fpls.2018.01548

**Published:** 2018-11-13

**Authors:** Jonathan J. Henn, Vanessa Buzzard, Brian J. Enquist, Aud H. Halbritter, Kari Klanderud, Brian S. Maitner, Sean T. Michaletz, Christine Pötsch, Lorah Seltzer, Richard J. Telford, Yan Yang, Li Zhang, Vigdis Vandvik

**Affiliations:** ^1^Department of Integrative Biology, University of Wisconsin-Madison, Madison, WI, United States; ^2^Department of Ecology and Evolutionary Biology, The University of Arizona, Tucson, AZ, United States; ^3^Department of Biological Sciences, University of Bergen, Bergen, Norway; ^4^Bjerknes Centre for Climate Research, University of Bergen, Bergen, Norway; ^5^Faculty of Environmental Sciences and Natural Resource Management, Norwegian University of Life Sciences, Ås, Norway; ^6^Department of Botany and Biodiversity Research Centre, The University of British Columbia, Vancouver, BC, Canada; ^7^Earth and Environmental Sciences Division, Los Alamos National Laboratory, Los Alamos, NM, United States; ^8^Institute of Mountain Hazards and Environment (CAS), Chengdu, China

**Keywords:** functional traits, phenotypic plasticity, alpine plants, climate change, intraspecific variation

## Abstract

In a rapidly changing climate, alpine plants may persist by adapting to new conditions. However, the rate at which the climate is changing might exceed the rate of adaptation through evolutionary processes in long-lived plants. Persistence may depend on phenotypic plasticity in morphology and physiology. Here we investigated patterns of leaf trait variation including leaf area, leaf thickness, specific leaf area, leaf dry matter content, leaf nutrients (C, N, P) and isotopes (δ^13^C and δ^15^N) across an elevation gradient on Gongga Mountain, Sichuan Province, China. We quantified inter- and intra-specific trait variation and the plasticity in leaf traits of selected species to experimental warming and cooling by using a reciprocal transplantation approach. We found substantial phenotypic plasticity in most functional traits where δ^15^N, leaf area, and leaf P showed greatest plasticity. These traits did not correspond with traits with the largest amount of intraspecific variation. Plasticity in leaf functional traits tended to enable plant populations to shift their trait values toward the mean values of a transplanted plants’ destination community, but only if that population started with very different trait values. These results suggest that leaf trait plasticity is an important mechanism for enabling plants to persist within communities and to better tolerate changing environmental conditions under climate change.

## Introduction

Understanding and predicting how plants will respond to accelerating climate change is paramount for conservation and the maintenance of ecosystem function (Chapin et al., 2000; Davis et al., 2005). Ecological theory suggests that plant functional traits (characteristics related to life history strategies) should be related to the ability of a species to survive and reproduce in a given set of environmental conditions (Funk et al., 2017). For example, the leaf economic spectrum is a set of leaf traits that characterize a continuum from fast to slow photosynthetic and tissue turnover rates (Wright et al., 2004; Reich, 2014; Díaz et al., 2016). Traits like those included in the leaf economics spectrum should be related to a species ability to persist under changing conditions. However, it has been difficult to test these predictions because variation in functional traits occurs at both inter- and intra-specific levels and can be affected by evolutionary history, environmental context, genetic constraints, and plasticity (Messier et al., 2010; Violle et al., 2012). Specifically, intraspecific trait variation can contribute approximately a quarter of the community trait variation in leaf and wood traits (Albert et al., 2010, 2012; Hulshof and Swenson, 2010; Jung et al., 2010; Messier et al., 2010; De Bello et al., 2011). This has also attracted attention as a potentially key component of community assembly (Hart et al., 2016; Des Roches et al., 2017; Hausch et al., 2018).

Intraspecific variation arises from both heritable differences and plasticity (Matesanz et al., 2012) and might be very important for adaptation in response to changing environmental conditions (Norberg et al., 2001; Björklund et al., 2009). For example, those traits that exhibit low variation in variable environments may respond slowly and lag behind shifting optimal trait values. This suggests that the relative extent of variation in a trait that is due to intraspecific variation as opposed to interspecific variation might be informative in predicting how species will respond to climate change (Dunne et al., 2003; Anderson and Gezon, 2015; Moran et al., 2015; Malyshev et al., 2016).

The distribution of traits within a community is expected to reflect variation around a mean optimal phenotype for fitness and/or growth rate (Norberg et al., 2001; Enquist et al., 2015). This idea follows from a central paradigm in ecology (Whittaker, 1972) and evolutionary biology (Levins, 1968) where observed shifts in phenotypes, species’ abundances, and composition across environmental gradients reflect the arrangement of phenotypes or species that maximizes fitness in different environments. Abiotic filters such as temperature or moisture that limit successful survival strategies promote convergence of traits around this optimal local phenotype (Keddy, 1992; Weiher and Keddy, 1999; Violle et al., 2012). On the other hand, when competitive interactions are a dominant factor in species survival or environmental conditions are highly variable at a scale smaller than the study unit, there could be advantages to expressing more variable phenotypes that are not clumped at the optimal phenotype (Pacala and Tilman, 1994; Grime, 2006). However, tests of this theory have shown mixed results with some evidence for higher fitness (Lajoie and Vellend, 2018) or occurrence (Muscarella and Uriarte, 2016) when species are nearer the community trait mean while other cases showed no tendency for species in their preferred habitats to have trait values closer to the community mean (Mitchell et al., 2018). The importance of intraspecific variation in this dynamic is also unclear.

Phenotypic plasticity can play a role in the persistence of individuals under changing climate conditions (Nicotra et al., 2010) but the extent of phenotypic plasticity can be limited by ecological and evolutionary constraints (Valladares et al., 2007). If phenotypic plasticity promotes a tendency to converge on community mean traits (Ghalambor et al., 2007), it could help predict the likely effects of climate change on plant communities. These patterns can be examined by transplanting communities and comparing phenotypes under different environmental conditions (Dunne et al., 2003; Anderson and Gezon, 2015; Guittar et al., 2016; Cui et al., 2018).

Here we use the results from a transplant experiment along an alpine elevation gradient on Gongga Mountain, Sichuan Province, China to provide a novel basis to assess how functional trait variation is structured and mediates responses to climate change. Specifically, we address three questions:

(1) For various plant functional traits, how does the amount of intraspecific variation compare to interspecific variation across an alpine elevation gradient?
(2) How do community functional trait distributions change across an alpine elevation gradient?
(3) When plants are transplanted to new environments, how plastic are their traits and does trait plasticity promote divergence or convergence toward community mean trait values?

To answer these questions, we measured leaf functional traits in a reciprocal transplant experiment across an elevation gradient where a transplantation down the gradient simulates a warmer climate (+1.5°C) and transplantation up the gradient simulates a cooler climate (-1.5°C). This amount of warming is consistent with the smallest projected changes for the next 20–40 years in nearby Himalayan regions (Wu et al., 2017). We conducted both warming and cooling treatments to examine the range of possible plant responses under contrasting environmental changes. As environmental conditions are changing rapidly in alpine environments, understanding the plasticity of leaf functional traits will help improve predictions of future plant community change.

## Materials and Methods

### Study Area and Experimental Setup

This study was situated in the Kang-Ding Valley of Gongga Mountain in the Hengduan Mountains in western Sichuan Province, China. The study region has a mean annual temperature of 11.6°C and mean annual precipitation of 800 mm (Fick and Hijmans, 2017). We studied plant communities at four sites along an elevation gradient: 3000 m (29.843469°, 102.034283°), 3500 m (29.86192°, 102.036°), 3800 m (29.88911°, 102.0173°), and 4130 m (29.85742°, 102.0118°) above sea level (Supplementary Figure S1). There is an approximately 1.5°C mean annual temperature difference between each of the sites. The vegetation at all sites is grassland characterized by a mix of subalpine and alpine species including grasses like *Festuca* spp. and *Poa* spp., sedges like *Carex* spp. and *Kobresia* spp., and forbs like *Anaphalis nepalensis, Clinopodium polycephalum*, and *Saussurea* spp. (Yang et al., in press). All study sites are on mountain gray-brown soil (He et al., 2005) and are grazed by yak, sheep, and horses. There is low variation in the timing of snowmelt along this gradient, as little snow falls during winter.

A whole-community turf transplant experiment was established at these sites in 2012 when exclosures were erected to prevent grazing (Supplementary Figure S1). Each transplanted turf measured 25 cm × 25 cm and were excavated to 20 cm depth or to where the soil ended, if that was shallower that 20 cm as was the case in the highest site. To control for the effect of transplantation, turfs were transplanted in the same site of origin. No differences were found between transplanted and untransplanted control plots, so both plot types were used as control plots in this study. Transplanted turfs were blocked such that each turf came from close to the control plots in the same block. In total, there were seven replicate turfs for each type of treatment and control at each site (except the highest site, where there are five replicates of each plot type, totaling of 92 plots used in this project). The local plant community composition and biomass was recorded in a separate set of 20, 50 cm × 50 cm plots (except for the highest site, where 13 plots were measured) at each site (for a total of 73 plots). These plots were used for characterizing the unmanipulated community composition at each site and are separate from the experimental transplant plots because biomass of each species was measured destructively in 2015. These community surveys were also used to characterize the regional species pool for partitioning of interspecific and intraspecific trait variation.

### Community Description

We collected leaves from the most common species in the plant community at each of the four sites in August 2015 and 2016. Leaves (*n* = 2,873) were collected from 164 species outside of the experimental plots and we aimed to collect one healthy, fully expanded leaf from up to five individuals for each species at each site where they occurred. However, this was not possible for all species at all sites. To avoid sampling clones, we selected individuals that were visibly separated from other stems of that species. Most species were sampled in only 1 year and species sampled over both years were unlikely to be from the same individuals, as the area from which we sampled was large. Numbers of leaves per site varied from 533 to 850 (L = 809, M = 867, A = 664, H = 533). All these leaves were used to assess leaf structural trait variance partitioning (LA, LT, SLA, and LDMC) while a subset of 209 observations from all sites combined were used to assess leaf nutrient and isotope variance partitioning. Between 57 and 85% of the biomass in the 50 cm × 50 cm plots used for community composition consisted of species with trait data, with between 97 and 100% of the biomass at each site comprised by genera with trait data (Supplementary Table S1). All taxa names were standardized using the Taxonomic Name Resolution Service (Boyle et al., 2013).

### Intraspecific Variation

Ten of the most common species along the gradient were selected for sampling in experimental plots during August 2016. These include *Artemisia flaccida, Epilobium fangii, Geranium pylzowianum, Hypericum wightianum, Pedicularis davidii, Persicaria vivipara, Plantago asiatica, Potentilla leuconota, Veronica szechuanica*, and *Viola biflora* var. *rockiana*. Of these species, only *P. leuconota* and *V. szechuanica* were present across the whole elevation gradient, so all other species were found in transplanted turfs outside of the elevation range where they are common. These species were selected to avoid species that readily spread clonally to prevent measuring individuals that did not originally occur on the experimental turf. Because many species in this system display at least some clonal reproduction and distinguishing genetic individuals is impossible without destructive sampling, we worked at the ramet level in each plot, as in Cui et al. (2018). We only selected species that were present in turfs prior to transplantation and that had remained present in transplanted turfs. For these species, up to five healthy, fully expanded leaves were collected from individuals in each experimental plot (i.e., control, locally transplanted control, warmed, and cooled) where that species occurred. A total of 2,246 leaves were collected and measured with 112–350 individuals per species to assess intraspecific variation for these 10 species.

### Functional Trait Measurements

We measured 11 functional traits related to potential physiological rates and environmental tolerance of plants. These include leaf area (LA, cm^2^), leaf thickness (LT, mm), leaf dry matter content (LDMC, g/g), specific leaf area (SLA, cm^2^/g), carbon (C, %), nitrogen (N, %), phosphorus (P, %), carbon:nitrogen (C:N), nitrogen:phosphorus (N:P), carbon13 isotope ratio (δ^13^C, ‰), and nitrogen15 isotope ratio (δ^15^N, ‰). Measurements were made based on standardized protocols from Pérez-Harguindeguy et al. (2013).

All leaves for trait measurements were collected and stored in plastic bags and coolers in the field before transport to the lab. At the lab, leaves were measured for leaf area, leaf thickness, and fresh mass. Leaf area was measured on Canon LiDE 220 scanners at 300 dpi. Following scanning, ImageJ (Schneider et al., 2012) and LeafArea package were used to calculate leaf area (Katabuchi, 2017). Leaf thickness was measured using calipers at three random locations on each leaf and the average taken for further analysis. Fresh mass was measured on a balance within 24 h of collecting leaves. Leaves were then dried for at least 72 h at 65°C before dry mass was measured. A subset of leaves was then ground into a fine powder and analyzed for nutrients and isotopes including P, N, C, δ^15^N, and δ^13^C at The University of Arizona. Total phosphorus concentration was determined using persulfate oxidation followed by the acid molybdate method (APHA, 1992). Phosphorus concentration was then measured colorimetrically with a spectrophotometer (ThermoScientific Genesys20, United States). Carbon, nitrogen, and their stable isotope ratios were measured by the Department of Geosciences Environmental Isotope Laboratory at The University of Arizona on a continuous-flow gas-ratio mass spectrometer (Finnigan Delta PlusXL) along with an elemental analyzer (Costech). Samples of 1.0 ± 0.2 mg were combusted and standardization was based on acetanilide for N and C concentration, NBS-22 and USGS-24 for δ^13^C, and IAEA-N-1 and IAEA-N-2 for δ^15^N. Ratios between C:N and N:P were also calculated and analyzed. Prior to analysis, samples with apparent measurement errors that resulted in unrealistic trait values were removed. This included leaves with leaf dry matter values higher than 1 g/g, leaves with specific leaf area values less than 5 cm^2^/g or greater than 500 cm^2^/g and leaf nitrogen values higher than 6.4%. The nitrogen cutoff values was chosen based on the highest published leaf nitrogen values found in the Botanical Information and Ecology Network (Enquist et al., 2009) for the genera in our study.

### Analyses

To quantify the extent of intraspecific vs. interspecific variation of leaf traits along the elevation gradient, we performed a variance partitioning analysis. This analysis assesses the variation in traits at different taxonomic levels (i.e., within-species, species, genus, family, and order) and between sites (populations) across the elevation gradient. We log transformed the data for the multiplicative growth traits (i.e., LA, LDMC, and LT) of all non-experimental leaves and performed a nested ANOVA using the lme (Bates et al., 2015) and varcomp (Qu, 2017) functions in R (Messier et al., 2010). For each level, the function first calculates the group mean. It then compares the variance around the group mean to the mean of the next level (e.g., variance of genus level is compared to the mean of family level). We used taxonomy as a substitute for phylogeny in the analysis. As a result, trait variance may be influenced by the loss of information about the ages of species, genera, families, and orders in relation to each other.

To describe community trait distributions at our sites, biomass-weighted community trait distributions were calculated for each site using non-parametric bootstrapping (Enquist et al., 2015, 2017). At each site we calculated 1000 replicate distributions. For each replicate, trait data for each species within the site was randomly sampled with replacement from the set of available trait data for that species. Trait data was not available for each species at each site, and so we prioritized trait data to use as follows: (1) focal species data from unmanipulated conditions at the focal site, (2) focal genus data from unmanipulated conditions at the focal site, (3) focal species data from any unmanipulated conditions, and (4) focal genera data from any unmanipulated conditions. When congener trait data were used as a proxy for focal species trait data, we randomly rarefied this data to ensure that each congener was equally likely to be sampled.

To examine phenotypic plasticity, we took the average trait value of each species in each plot to make comparisons between the trait values of plants in plots that had been transplanted with plants in plots that hadn’t been moved. We only compared plots from the same block to reduce variation due to microtopographic and microclimatic variation at each site. First, we assessed if the experimental treatment influenced the relative plasticity of each trait, *P_R_* by calculating the extent to which the traits of the plants in transplanted plots (T) changed with respect to plants in plots in their original home (H). This is a simplified Relative Distance Plasticity Index (Valladares et al., 2006), which is useful for comparing plasticity of traits or species under different environmental conditions and was done by calculating:

$$P_{R}=||(H-T)|/H|$$

where H is the mean trait value of all individuals within the home control plot and T is the mean trait value of all individuals within the transplanted turf. The absolute value of the numerator is taken to standardize all potential trait shifts while the absolute value of the whole quotient is to standardize trait values that are negative (δ^13^C). Low values of *P_R_* indicate that the observed intraspecific variation in each trait in an experimental plot shows no change in trait mean relative to the home populations. In contrast, high values of *P_R_* indicate a change in intraspecific mean trait value induced by the experiment. We calculated *P_R_* for each species where we had paired observations between their home (H) and transplanted (T) site. We tested whether traits differ in their response to transplantation by using linear mixed effect models where *P_R_* was modeled as a function of trait, transplant type (warming vs. cooling), and their interaction with species and site as random effects to account for multiple samples from each species and site. We used the lmer function with Satterthwaite estimations for degrees of freedom for hypothesis testing from the lmerTest R package (Kuznetsova et al., 2017).

Next, to assess whether transplanted plants shifted their functional traits toward the community mean of their transplant community, we assigned observations to several binary groups including:

(1) Whether the trait value of plants converged on, or diverged from, the destination site community trait means after transplantation. **“Converging”** corresponds to plants whose trait values moved closer to their transplantation destination community trait mean after transplantation. In contrast, **“Diverging”** corresponds to plants whose trait values moved further from their destination community trait mean after transplantation.
(2) Whether the transplant was to higher or lower temperatures. **“Warming”** corresponds to transplants to higher temperatures (+ ∼1.5°C) and **“Cooling”** corresponds to transplants to lower temperatures (-∼1.5°C).
(3) Whether the trait values of plants were closer to their home community trait mean or the transplant destination community trait mean prior to transplantation. **“Home”** corresponds to plants who started with trait values closer to their home community mean value (further from their destination mean trait value) and **“Destination”** corresponds to plants who started with trait values closer to the transplant destination community mean value (further from their home mean trait value).

We used log-likelihood ratio tests (G-tests, Signorell and Al et mult, 2018) to determine whether the proportion of comparisons converging or diverging (category 1 above) is dependent on transplant type (category 2 above) and whether the trait value started closer to the home or destination community values (category 3 above) for all traits together. We also determined whether transplantation induced a significant response in trait values by calculating the 99% confidence interval assuming a t-distribution around the mean value of each trait for each species under unmanipulated conditions at each site. If the mean value of a species trait value in a transplanted site fell outside of the 99% confidence interval from its home site, then it was considered a significant plastic response at the *p* < 0.01 level. All analyses were conducted in R 3.4.2 (R Core Team, 2017).

## Results

### Intraspecific Variation

**FIGURE 1:**
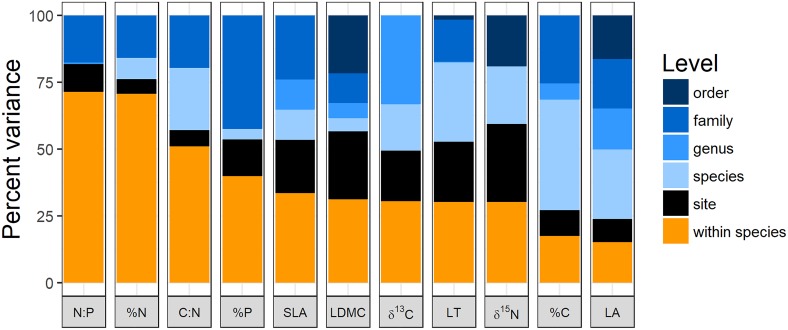
Variance partitioning of leaf traits at different taxonomic levels and between populations across sites along the elevation gradient. LA, leaf area; LT, leaf thickness; LDMC, leaf dry matter content; SLA, specific leaf area; %C, % carbon content of leaves; %N, % nitrogen content of leaves; %P, % phosphorus content of leaves; C:N, carbon:nitrogen; N:P, nitrogen:phosphorus; δ^13^C, carbon 13 isotope ratio; δ^15^N, nitrogen 15 isotope ratio. Data for LDMC, LT, and LA were log transformed prior to analysis.

The variance partitioning analysis shows that nitrogen-related leaf traits (except for δ^15^N) tended to have very high intraspecific variation with >75% of variation being found within species or sites (Figure 1). A set of other traits including specific leaf area, leaf dry matter content, leaf thickness, %P, and isotope rations had intermediate intraspecific variation, ranging from 25 to 40% of variation. Finally, %C and leaf area had less than 25% of their variation at the intraspecific level (Figure 1).

### Traits Along Gradient

**FIGURE 2:**
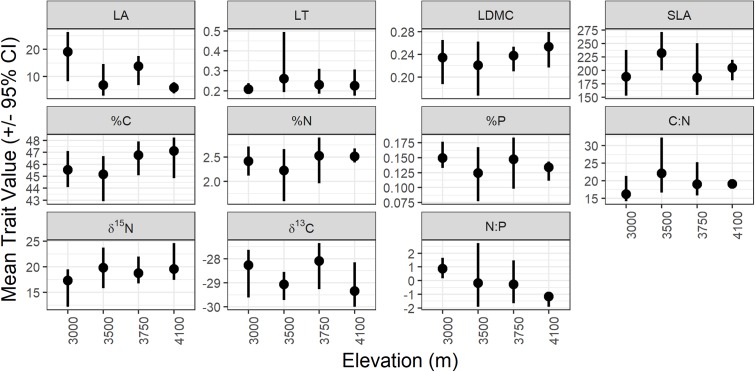
Bootstrapped mean (dot) and 95% confidence interval (line) for each trait at each study site. LA, leaf area (cm^2^); LT, leaf thickness (mm); LDMC, leaf dry matter content (g/g); SLA, specific leaf area (cm^2^/g); %C, % carbon content of leaves (%); %N, % nitrogen content of leaves (%); %P, % phosphorus content of leaves (%); C:N, carbon:nitrogen; N:P, nitrogen:phosphorus; δ^13^C, carbon 13 isotope ratio (‰); δ^15^N, nitrogen 15 isotope ratio (‰).

All traits varied between sites across the elevation gradient, but few had directional shifts along the elevation gradient (Figure 2). While there is substantial overlap in trait variation in many of the traits across the elevation gradient (overlap in the confidence intervals for all traits between sites, indicating few significant differences in mean trait values between elevations) there are some consistent shifts in the community biomass-weighted mean trait values with elevation.

### Phenotypic Plasticity

**FIGURE 3:**
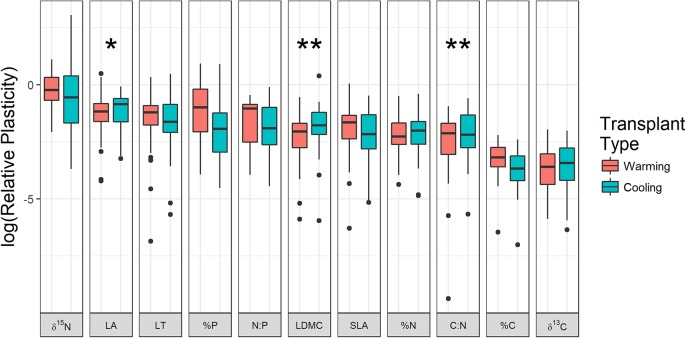
Log-transformed relative plasticity of each trait split by warming and cooling transplant treatments. Each observation represents the mean change in trait values for all individuals moved in each transplanted turf. Asterisks indicate traits where plasticity was significantly greater in cooling transplants compared to warming transplants (^∗^*p* < 0.1, ^∗∗^*p* < 0.05). LA, leaf area; LT, leaf thickness; LDMC, leaf dry matter content; SLA, specific leaf area; %C, % carbon content of leaves; %N, % nitrogen content of leaves; %P, % phosphorus content of leaves; C:N, carbon:nitrogen; N:P, nitrogen:phosphorus; δ^13^C, carbon 13 isotope ratio; δ^15^N, nitrogen 15 isotope ratio.

Phenotypic plasticity varied substantially by trait where δ^15^N, leaf area, and %P were most plastic while C and δ^13^C were least plastic in response to transplantation (Figure 3). For three traits, the amount of phenotypic plasticity varied by transplant treatment, where C:N (df = 704.6, *t* = 2.41, *p* = 0.016), leaf area (df = 708.4, *t* = 1.90, *p* = 0.058), and leaf dry matter content (df = 708.4, *t* = 2.56, *p* = 0.011) all tended to change more when transplanted to cooler locations compared to warmer locations (Figure 3). Species and origin site random effects both had significant variance (species: χ^2^ = 13.9, df = 1, *p* < 0.001; origin: χ^2^ = 10.3, df = 1, *p* = 0.001) indicating that phenotypic plasticity varied by species and by origin site.

**FIGURE 4:**
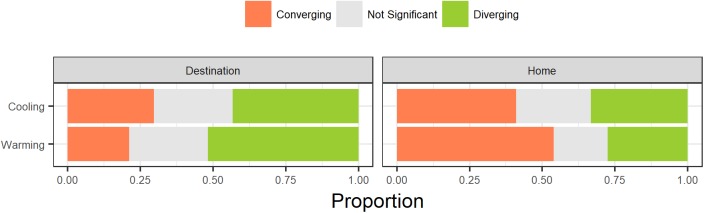
Proportions of observations where transplanted plant functional trait values converged or diverged relative to community mean trait values in transplant destination communities. Rows correspond to warming and cooling treatments. Columns correspond to whether the trait value of a species was closer to its **destination** community trait mean or the **home** community trait mean prior to transplantation. Gray colors indicate comparisons where trait values of transplanted plants fell within the 99% confidence interval of the trait values for untransplanted plants in their home (no significant difference, i.e., no plastic response).

Slightly more transplants showed divergence in their trait value from the destination community mean after transplantation (*n* = 279) than transplants that showed convergence toward their destination community mean (*n* = 277) while 180 transplants did not significantly change (Figure 4). Additionally, slightly more transplants had functional trait values closer to their home community prior to transplantation (*n* = 400) compared to transplants where functional trait values were closer to their destination community prior to transplantation (*n* = 336). When combined, individuals were more likely to converge with their destination community trait mean if they started further away from their destination community mean (Figure 4, *G* = 36.54, df = 2, *p* < 0.001). Warming transplants were slightly more likely to result in convergence while cooling transplants were slightly more likely to result in divergence but this was not a significant difference (*G* = 2.49, df = 2, *p* = 0.29). Between traits, the proportions of individuals converging and diverging varied substantially and this depended on both the type of transplant (warming or cooling) and where the individual trait value started relative to their destination trait community mean (Supplementary Figure S2). Foliar %C showed consistently high rates of convergence under all conditions except when transplanted plants were warmed and they started closer to their home trait values. Those traits that showed greatest convergence did not tend to be those traits that varied more in community biomass-weighted mean values along the elevation gradient.

## Discussion

Phenotypic plasticity will likely be a very important way for organisms to tolerate changing future climate conditions (Nicotra et al., 2010; Valladares et al., 2014). The combination of a transplant experiment and an elevation gradient have allowed us to examine the extent and directionality of phenotypic plasticity and intraspecific trait variation under simulated climate change. This experiment reveals that all traits studied showed some level of plasticity in response to changing climate and a general trend of convergence toward the trait mean of their new community when that mean was far from their home trait value.

### Intraspecific Variation and Phenotypic Plasticity

There was little evidence that traits that have more intraspecific variation at the regional scale also have greater plasticity in response to climate manipulations. The structural leaf trait with most intraspecific variation (SLA) showed moderate plasticity while the trait with lowest intraspecific variation (leaf area) showed high plasticity (Figures 1, 3). Nitrogen-related traits with high intraspecific variation also tended to have moderate plasticity. Trait variation in the species pool involves long-term community assembly processes that are generating or constraining variation over long time-scales and is likely subject to different constraints compared to phenotypic plasticity (Zobel, 1992). On these long time-scales there are costs and benefits to having high intraspecific variation (Valladares et al., 2007). Our measure of phenotypic plasticity, on the other hand, is a response to rapid change on the scale of years where only the individuals who can respond quickly can survive. Second, there are only certain traits that are likely to respond to the types of environmental change imposed on the transplanted plants in our study. For example, with decreasing mean temperature, there was an overall shift toward dominance by more conservative strategies (lower leaf area, higher thickness and leaf dry matter content, increased %C content, and lower %N). Further, colder sites are more dominated by plants with lower foliar N:P and δ^15^N. Specific leaf area, on the other hand, showed no directional change along the gradient, indicating that other factors than temperature are the most important determinants of optimal SLA values. These factors could include other abiotic conditions like differences in growing season length or biotic conditions like differences in soil microbe communities between sites (Dunne et al., 2003; Tomiolo et al., 2015). Finally, our calculations of inter- versus intra-specific trait variation did not include measurements from transplanted individuals because only a subset of species were transplanted. If these transplanted individuals were included, we would expect much higher measures of intraspecific variation, as phenotypic plasticity can substantially weaken phylogenetic signal in functional traits (Burns and Strauss, 2012).

### Extent of Phenotypic Plasticity

The functional traits that showed the most plasticity, including leaf area and leaf thickness, are both related to leaf lifespan, where thicker and smaller leaves have higher leaf mass per area and are thus more likely to have longer leaf lifespans and more conservative strategies (Wright et al., 2004; Reich, 2014). The observed plasticity in these traits could be driven by the imposition of additional stress when transplanted to colder climates, or the release of that stress when transplanted to warmer climates, as there is some tendency toward more acquisitive leaf strategies at lower elevations (Cornwell and Ackerly, 2009; Read et al., 2014). However, we found that the extent and importance of plasticity in traits varies by species. This is similar to Cui et al. (2018), who found that *Viola biflora* var. *rockiana* had low plasticity in leaf area in response to transplantation in the same study system. Plasticity in leaf P is likely to be more related to P availability in the soil along the elevation gradient. Leaf P may be less genetically controlled compared to environmentally controlled by access to that nutrient so movement to new places results in different P availability. δ^15^N also had high plasticity, potentially for similar reasons to %P. Variation in foliar δ^15^N has been linked to variation in soil N supply and nitrogen sources are primarily determined by local differences in N fixation, uptake, and outside sources (Craine et al., 2015) which could vary substantially between sites. Carbon-related traits (%C and δ^13^C) showed the lowest degree of plasticity in response to transplantation. This indicates that these traits might be more genetically controlled with little response to the environmental conditions in this experiment. Little variation in δ^13^C suggests that moisture conditions do not vary substantially between transplant sites, resulting in little change in isotope ratios (or water use efficiency) between sites.

It is important to note that while we refer to trait shifts with transplantation as plastic, our comparisons rely on the assumption that transplanted plants started with similar phenotypes to the other plants at the site where they originated. This means that we cannot rule out the importance of maternal effects and epigenetic inheritance. Additionally, the observed phenotypic plasticity in this study may or may not be adaptive and the extent to which phenotypic plasticity is adaptive is difficult to quantify (van Kleunen and Fischer, 2005; Ghalambor et al., 2007).

### Trait Convergence and Divergence

According to theory (Enquist et al., 2015), phenotypic plasticity may be adaptive if it results in movement toward a trait optimum that is adaptive for a specific environment. We did find evidence that transplanting to a new community results in a plastic shift in trait values toward the new community dominant trait value. This was especially true when the transplanted species differed more from the members of its new community before transplantation. This central tendency is consistent with the results found by Muscarella and Uriarte (2016) and Lajoie and Vellend (2018). However, when the difference between the transplanted species and the community mean trait value was small, phenotypic plasticity tended to result in divergence from the community mean. This could be a signature of biotic interactions where occupying space away from the mean trait value could be advantageous. On average, convergence was more common when transplants were moved to warmer conditions, especially when those plants started far from their destination community. This could be because competition is limiting the extent of higher elevation species at lower elevations and that this filter is stronger than the lower temperatures at higher elevations. Species moving to lower elevations can only survive if they can express the most competitive phenotype under these warming conditions.

Observations of convergence and divergence as plastic response to climate change could be due to other factors that we could not measure in this study. For example, phenotypic plasticity can be expressed as a result in interaction with new neighbors (Lipowsky et al., 2015; Abakumova et al., 2016) and transplanted plants were exposed to changing communities through time (Yang et al., in press). Our study also did not fully address the potential for fine-scale niche partitioning within communities. Such small-scale processes may be an important mechanism for the maintenance of local functional diversity (Stark et al., 2017). For example, microclimatic environmental conditions can vary within sites including soil depth, chemistry, water availability, light variation, and exposure to sun. Further, we did not assess multivariate shifts in traits between populations which may also better reveal the multivariate nature of community assembly (see Kraft et al., 2015). As climate continues to change, the limits of this plasticity will be important to consider, but this study demonstrates the importance of considering both a species’ traits and the plasticity in those traits when considering their ability to tolerate climate change.

If environmental controls on community assembly were strong along the gradient we would predict more consistent patterns of trait convergence toward community means. In our study site, like many other gradient studies (Wright et al., 2004; Muscarella and Uriarte, 2016), there is substantial overlap between mean trait values from site to site in most trait values (Figure 2). If there were substantial or directional trait differences between each site, those difference are likely to be driven mostly by species turnover. Under that scenario, transplantation would likely result in death, and only the most extreme plastic responses would promote survival. Since we are only working with species who have survived in their new conditions after transplantation for 5 years, all the intraspecific variation and plastic responses represent relatively successful strategies. We are not able to assess whether the individuals that did not survive had lower phenotypic plasticity or different tendencies than the winners reported here, but Guittar et al. (2016) found that community trait values responded to transplantation by converging toward local trait values over time as species composition changed.

## Conclusion

A trait-based approach to community ecology is providing valuable insight into both the physiological mechanisms underpinning species’ broad-scale geographical distributions and patterns of local diversity (McGill et al., 2006). Indeed, resolving patterns of trait–environment relationships and intra- and interspecific trait variation is critical for developing predictive models in community ecology (Laughlin et al., 2012; Violle et al., 2012). The ability of a species to adjust its phenotype as the climate changes rapidly will be very important in plant persistence under new conditions. This is especially important in cold biomes, where climate is changing most rapidly.

We assessed if the traits that are more variable will respond more quickly to environmental change. Our results show that a variety of alpine species had substantial phenotypic plasticity, although this plasticity was not necessarily related to intraspecific variation of these traits. Traits with high intraspecific variation did not correspond to traits that showed the highest plasticity in response to transplantation. We also assessed if patterns of intraspecific variation supported either community ecology models of intraspecific trait convergence or divergence. On the one hand, our results provide support of the community-weighted mean optimality hypothesis and support the assumption often made in community ecology that trends in intraspecific trait variation tend to mirror trends in interspecific variation (Muscarella and Uriarte, 2016). Specifically, when species are moved into new climates, traits tend to shift toward local optima. This suggests that there is some advantage to adopting a similar phenotype to other species, but only if a plants’ phenotype started different to the members of its new community. On the other hand, our results also support trait divergence hypotheses (Pacala and Tilman, 1994; Valladares et al., 2007), but only if traits from the transplanted population are already close to the new community mean trait value. These seemingly contradictory results indicate that hypotheses of trait convergence and divergence may not be mutually exclusive and instead are dependent on the context of the underlying processes (Grime, 2006). Together, our results indicate that trait plasticity is an important mechanism for enabling plant populations to persist within communities and to better tolerate changing environmental conditions under climate change.

## Author Contributions

VV and KK designed the transplant experiment. YY secured funding and implemented the experiment. VB, JH, BE, KK, CP, SM, VV, and YY designed and implemented the field sampling design. BM, KK, JH, LS, CP, and LZ collected plant trait data. LZ collected community composition and biomass data. AH, CP, and RT cleaned and processed the data. BM, JH, and LS conducted analyses. JH wrote the manuscript with input from BE, BM, and VV. All authors have commented on and approved the final manuscript. Author order is first-last-emphasis with alphabetical order in the middle.

## Conflict of Interest Statement

The authors declare that the research was conducted in the absence of any commercial or financial relationships that could be construed as a potential conflict of interest. The reviewer CD declared a shared affiliation, though no other collaboration, with one of the authors SM to the handling Editor.
