# Supplementary material for: Intraspecific Trait Variation and Phenotypic Plasticity Mediate Alpine Plant Species Response to Climate Change
Source: Front Plant Sci. 2018 Nov 13;9:1548. doi: 10.3389/fpls.2018.01548 (PMC6243391; doi:10.3389/fpls.2018.01548)
Supplement: Supplementary file 1 [file Data_Sheet_1.docx]

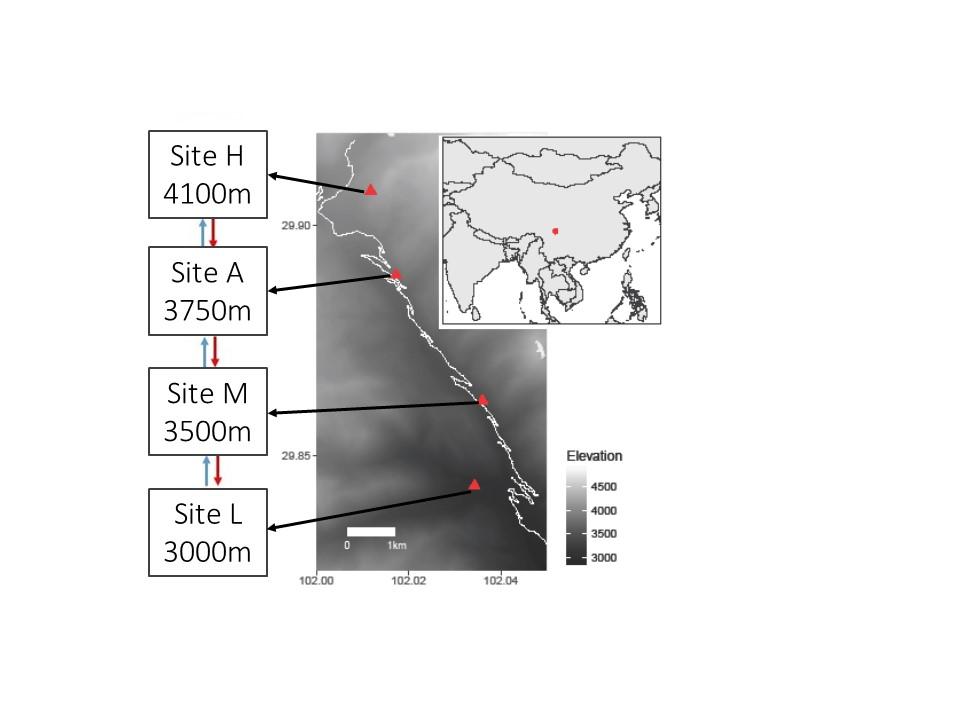


**Figure S1:** Map of the location of study sites in Sichuan province, China. Red arrows indicate warming transplants while blue arrows indicate cooling transplants.

**
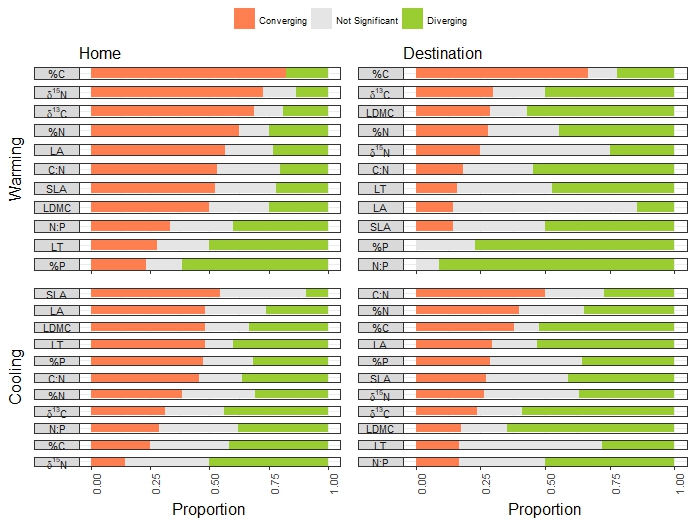
**

**Figure S2:** Proportions of observations where transplanted plant functional trait values converged with or diverged from community mean trait values in transplant destination communities for all traits. Columns correspond to whether the trait value of a species was closer to its **destination** community trait mean or the **home** community trait mean prior to transplantation. Rows correspond to whether a transplant was to a **warmer** or **cooler** environment. Gray colors indicate comparisons where trait values of transplanted plants fell within the 99% confidence interval of the trait values for untransplanted plants in their home (no significant difference, i.e., no plastic response). LA = leaf area, LT = leaf thickness, LDMC = leaf dry matter content, SLA = specific leaf area, %C = % Carbon content of leaves, %N = % Nitrogen content of leaves, %P = % Phosphorus content of leaves, C:N = Carbon:Nitrogen, N:P = Nitrogen:Phosphorus, δ^13^C = Carbon 13 isotope ratio, δ^15^N = Nitrogen 15 isotope ratio.

**Table S1:** Percentages of species-level and genus-level traits that were included in community trait distribution bootstrapping.

| **Site (m)** | **Spp w/ data (%)** | **Genera w/ data (%)** |
| --- | --- | --- |
| **4130** | 81.39 | 97.28 |
| **3800** | 57.11 | 97.15 |
| **3500** | 59.97 | 97.70 |
| **3000** | 85.15 | 99.96 |
